# Glycemic Profile and Lipid Profile in Chronic Obstructive Pulmonary Disease (COPD) Patients With and Without Metabolic Syndrome

**DOI:** 10.7759/cureus.58921

**Published:** 2024-04-24

**Authors:** Binod Mahato, Shreya Nigoskar, Lingidi Jhansi Lakshmi, Doddigarla Zephy

**Affiliations:** 1 Department of Biochemistry, Index Medical College & Research Center, Malwanchal University, Indore, IND; 2 Department of Biochemistry, Hi-Tech Medical College & Hospital, Bhubaneswar, IND; 3 Department of Biochemistry, Hi-Tech Medical College & Hospital, Rourkela, IND

**Keywords:** high-density lipoproteins, emphysema, triglycerides, mean blood glucose, impaired fasting glucose, chronic obstructive pulmonary disease

## Abstract

Objectives: There is a lack of Indian data regarding the frequency of metabolic syndrome (MetS) or its components in chronic obstructive pulmonary disease (COPD). As a result, the present study aimed to determine the prevalence of MetS in COPD cases and investigate its association with COPD severity.

Material: After receiving ethical approval from Index Medical College and Hospital, we conducted this cross-sectional study in Indore. We recruited 100 participants with a history of COPD and divided them into two groups: those with MetS and those without. Researchers examined the subjects' fasting blood glucose, serum high-density lipoprotein, triglyceride (TG), systolic and diastolic blood pressure (SBP/DBP), waist circumference, and fasting blood glucose levels.

Results: We discovered that 59% of patients with COPD and 52% of individuals with impaired fasting glucose (IFG) had MetS (mean ± SD = 110.8 ± 32.8). In comparison, 48% (mean ± SD = 98.2 ± 24.8) of individuals with normal fasting glucose do not experience this. The incidence of MetS was higher in both groups, those with IFG and those without, but the difference was not statistically significant (t = 1.7088, df = 98; p = 0.0907). We observed X2 = 1.336, df = 1, and p = 0.2476 when we tested the association between IFG and COPD with the Chi-square test.

Conclusion: Individuals with MetS were more likely to have high BP, raised TG levels, low HDL cholesterol, abdominal obesity, and other risk factors.

## Introduction

Chronic obstructive pulmonary disease (COPD) is a significant contributor to the overall burden of healthcare worldwide. It is also the sole leading cause of mortality that is experiencing a rise in prevalence [1]. COPD is a multifaceted and diverse disorder with notable manifestations outside the lungs, such as cardiovascular disease, skeletal muscle weakness, and diabetes [2]. COPD is associated with a range of systemic symptoms. Researchers have acknowledged the coexistence of metabolic syndrome (MetS) with COPD [2]. MetS is commonly characterized by a combination of five factors: elevated blood pressure (BP), increased triglyceride (TG) levels, reduced levels of high-density lipoprotein cholesterol (HDL), excess belly fat, and elevated glucose levels [3]. Reaven first documented the condition in 1988, alternatively referring to it as insulin resistance syndrome or syndrome X [4]. Multiple studies have discovered a connection between MetS and COPD. Researchers have recognized MetS as a separate risk factor for exacerbating respiratory symptoms, worsening lung function impairment, causing pulmonary hypertension, and triggering asthma [5]. Researchers have hypothesized that obesity, smoking, a sedentary lifestyle, and systemic inflammation contribute to its development [2]. COPD patients with comorbid MetS have more pronounced systemic inflammation compared to patients with COPD alone [6]. The incidence of MetS in people with COPD is estimated to be between 21% and 53% [2]. There is a lack of Indian data regarding the frequency of MetS or its components in COPD. Dave and colleagues [7] found a prevalence of MetS in 42% of their patients with COPD compared to only 20% of controls of the same age. MetS patients with COPD are more likely to experience dyspnea, breathing difficulties, and hospitalization [7]. Acute exacerbations of the condition, or secondary complications, can result in hospitalization. In India, the majority of COPD patients often receive their first treatment from primary physicians. General physicians' ability to identify MetS at the primary level can decrease hospitalization risk. Smoking, the primary determinant for COPD development, is considered a primary catalyst for heightened systemic inflammation, thereby elucidating the association between MetS and COPD [8]. Systemic inflammation exacerbates insulin resistance, thereby playing a role in the pathogenesis of MetS in people with COPD [9]. A study found that MetS is more common in female patients and people with less severe COPD and a high body mass index (BMI). The present study aimed to determine the prevalence of MetS in cases of COPD and investigate its association with the severity of COPD [10]. This study aims to assist primary physicians in identifying MetS components in patients with COPD at an early stage. The findings will enable physicians to tailor care strategies based on specific patient needs.

## Materials and methods

This observational study used a cross-sectional design involving 100 individuals with MetS visiting Index Medical College and Hospital six months after obtaining ethical approval under the reference of MU/Research/EC/Ph.D/2021/.

Inclusion and exclusion criteria

We used the global initiative for COPD (GOLD) recommendations to diagnose patients with COPD after carefully evaluating their pulmonary function test, clinical examination, and medical history. The study excluded patients with cancer, active pulmonary tuberculosis, asthma, other chronic respiratory conditions, major comorbidities, and those who had used systemic corticosteroids within the previous three months.

Evaluation of COPD

We used the global initiative for COPD (GOLD) recommendations to diagnose patients with COPD after carefully evaluating their pulmonary function test, clinical examination, and medical history. The study excluded patients with cancer, active pulmonary tuberculosis, asthma, other chronic respiratory conditions, and major comorbidities and those who had used systemic corticosteroids within the previous three months. We asked patients to complete a survey in their native tongue, collecting their responses with the utmost respect for privacy and confidentiality. We collected information on lifestyle factors (smoking behavior and exercise) and demographic characteristics (age, gender, ethnicity, education, occupation, and monthly income) in a transparent and easily understandable manner. We performed every standard examination, which included a lipid profile, fasting, and two-hour postmeal blood sugar level measurements (FBS and 2-h PPBS, respectively). Before the research began, we provided each participant with comprehensive information about the procedure and obtained their written consent. We collected the anthropometric data, which included the subjects' height, weight, and waist circumference, using appropriate methodologies. We determined the standing body height to an accuracy of 0.5 cm using a commercial stadiometer. The accuracy of the computerized weighing machine (± 100 g) was utilized in the determination of body weight (BW). 

Evaluation of MetS

Researchers evaluated the subjects' fasting blood glucose, serum HDL, TG levels, SBP/DBP, waist circumference, and fasting blood glucose levels to determine the presence of MetS. Each participant underwent two measurements of SBP and DBP, separated by three minutes, following 15 minutes of rest. We then computed the average SBP and DBP for analysis. We assessed the fasting blood glucose and TG levels in the morning, following an 8-12-hour fast without consuming any sustenance. We measured the distance across the highest lateral border of the right ileum at the end of the subject's typical exhalation to determine the waist circumference. We used the National Cholesterol Education Program Adult Treatment Panel-III (NCEP ATP-III) established criteria for diagnosing MetS. According to this definition, the presence of three or more of the following elements confirms the existence of MetS: Abdominal obesity is defined by a minimum waist circumference of 90 cm in males and 80 cm in females. Additionally, we regard elevated TG levels (150 mg/dL or higher) or the administration of medication to control such levels as indicators of this condition. Individuals who meet the following criteria: men with HDL levels below 40 mg/dL; women with HDL levels below 50 mg/dL; individuals undergoing HDL reduction medication; those with systolic blood pressure (SBP) or diastolic blood pressure (DBP) exceeding 130 mm Hg or 85 mm Hg, respectively; individuals with FBS levels equal to or exceeding 100 mg/dL; or those who are taking medication to manage hypertension [10,11].

Statistical analysis

We conducted the statistical analyses using the IBM SPSS Statistics for Windows, Version 20 (Released 2011; IBM Corp., Armonk, New York, United States). We used percentages to represent the categorical variables and the mean and standard deviation to report the continuous variables. We utilized the Chi-square test to determine the correlations between category variables. We assessed group differences in means for continuous data using the unpaired Student's t-test and employed the Chi-square test for categorical data. A statistically significant threshold of p < 0.05 was established.

## Results

In our study, the average waist-to-hip ratio is 0.98 ± 0.02. The average SBP/DBP in our study (Table 1) was 138.4 ± 16.1 and 91 ± 10.2 mmHg, respectively. On average, FBS is 102.4 ± 16.3 mg/dl. The mean total cholesterol (Tc) and TG values are 164.2 ± 43.2 and 168.2 ± 44.3 mg/dl, respectively. Our population has mean HDL, LDL, and VLDL cholesterol readings of 43.1, 94.2, and 39.2 mg/dl, respectively.

**Table 1:** Characteristics of the study population FBS: Fasting blood sugar; Tc: total cholesterol; HDL: high-density lipoprotein; LDL: low-density lipoprotein; VLDL: very low-density lipoprotein; TG: triglyceride Data presented in mean and standard deviation

Variable	Total	Mean ± standard deviation
FBS (mg/dl)	100	102.4 ± 16.3
Tc (mg/dl)	100	168.2 ± 44.3
HDL (mg/dl)	100	43.1 ± 6.5
LDL (mg/dl)	100	94.2 ± 46.4
TG (mg/dl)	100	164.2 ± 43.2
VLDL (md/dl)	100	39.2 ± 6.9

**Figure 1:**
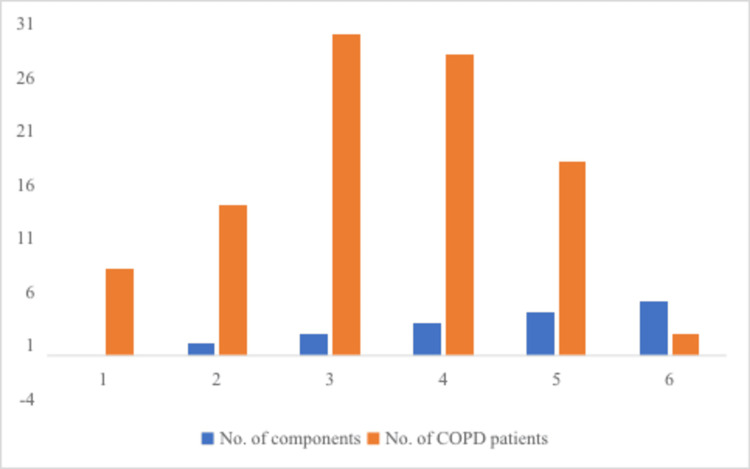
Distribution of MetS components in COPD patients Data presented in percent

Only two of the participants in our study (Figure 1) were found to have all five of the MetS components, while the other seven individuals had none of them at all. Only 14 patients had only one component of the MetS, whereas 30 people had two components of the MetS. The 46 people who had either one or both of the components of MetS had an increased risk of developing cardiovascular disease and are more likely to go on to develop MetS in the future. As a direct consequence of this, these people need MetS screening.

We discovered that 59% of patients with COPD and 52% of individuals with impaired fasting glucose (IFG) had MetS (mean ± SD = 110.8 ± 32.8) (Table 2). In comparison, 48% (mean ± SD = 98.2 ± 24.8) of individuals with normal fasting glucose do not experience this. The incidence of MetS was higher in both groups, those with IFG and those without, but the difference was not statistically significant (t = 1.7088, df = 98; p = 0.0907). When the association between IFG and COPD was tested with the Chi-square test, we observed X2 = 1.336; df = 1; and p = 0.2476.

**Table 2:** Impaired fasting glucose (IFG) and metabolic syndrome (MetS) in the study population Data presented in mean and standard deviations. p < 0.05 considered for statistical significance

IFG	MetS patients with COPD	Non-MetS patients with COPD	Mean ± SD	p-value
Yes	26	26	110.8 ± 32.8	= 0.0907
No	18	30	98.2 ± 24.8	

Table 3 shows patients with MetS and abdominal obesity from the study population. About 55% of the individuals in our study were not obese in the abdomen. Thirteen individuals with COPD or MetS did not exhibit any signs of abdominal obesity. Forty-five patients with COPD who were abdominally obese had a higher prevalence of MetS than those who were not.

**Table 3:** Abdominal obesity and metabolic syndrome (MetS) patients in the study population: MetS patients with COPD N and non-MetS patients with COPD N MetS:  Metabolic syndrome Data presented in mean and standard deviations. p < 0.05 considered for statistical significance

Abdominal obesity	MetS patients with COPD N	Non-MetS patients with COPD N
Yes	31	14
No	13	42

Table 4 displays the number of research group members with BMIs greater than 25 kg/m2 who suffer from MetS. According to these results, a total of 45 patients were overweight in the present study (mean ± SD = 26.85 ± 6), of which 28 COPD patients had MetS who were overweight (mean ± SD = 27.3 ± 5.8) and overweight seventeen non-MetS patients with COPD had a BMI of (mean ± SD = 26.4 ± 6.2). When compared, there was no statistical difference (t = 0.4918; df = 43; P = 0.6254). Out of 55 non-overweight patients (mean ± SD = 23.1 ± 5.5), only sixteen people with BMIs under 25 (mean ± SD = 23.4 ± 5.8) were identified as having MetS, and 39 patients had COPD with no MetS (mean ± SD = 22.8 ± 5.2), nevertheless. Among COPD patients who had a BMI of ≥ 25 kg/m2 and BMI < 25 kg/m2, when compared, the statistical difference was highly significant (t = 3.2559; df = 98; P = 0.0016). We tested the association between BMI and COPD with the Chi-square test, observing X2 = 11.0258; df = 1; and P = 0.0008.

**Table 4:** Body mass index (BMI) ≥ 25 Kg/m2 and metabolic syndrome (MetS) patients in the study population Data presented in mean and standard deviations. p < 0.05 considered for statistical significance

Body Mass Index ≥ 25 kg/m^2^	MetS patients with COPD Mean ± SD	Non-MetS patients with COPD Mean ± SD	Total Mean ± SD	P Value
Yes	27.3 ± 5.8	26.4 ± 6.2	26.85 ± 6	0.0016
No	23.4 ± 5.8	22.8 ± 5.2	23.1 ± 5.5	

Table 5 displays the number of TG-afflicted research group members with MetS and those without it. According to these results, 42 patients showed a TG ≥ 150 mg/dl (mean ± SD = 175.85 ± 21.65). Out of 42 patients, 30 showed MetS with COPD with TG (mean ± SD = 178.8 ± 23.8), and 12 patients showed COPD but no MetS with TG values (mean ± SD = 172.9 ± 19.5). When compared between these two, we did not observe a statistical difference (t = 0.8662; df = 40; p = 0.3916). About 58 patients in the present study showed TG levels < 150 mg/dl (mean ± SD = 127.6 ± 16.2), of which 14 had MetS with COPD with a mean TG value of (mean ± SD = 127.5 ± 15.8) and 44 patients had COPD with no MetS with a TG level (mean ± SD = 128.1 ± 16.7). Among COPD patients who had a TG of ≥ 150 mg/dl when compared with patients with a TG < 150 mg/dl, the statistical difference was highly significant (t = 12.7522; df = 98; p < 0.0001). Using the Chi-square test to test the association between TAG and COPD, we observed X2 = 18.3476, df = 1, and p > 0.05.

**Table 5:** Triglycerides (TG) and metabolic syndrome (MetS) patients in the study population Data presented in mean and standard deviations. p < 0.05 considered for statistical significance

TG ≥ 150 mg/dl	MetS patients with COPD Mean ± SD	Non-MetS patients with COPD (mean ± SD)	Total mean ± SD	p-value
Yes	178.8 ± 23.8	172.9 ± 19.5	175.85 ± 21.65	<0.0001
No	127.5 ± 15.8	128.1 ± 16.7	127.6 ± 16.2	

Table 6 displays the number of research group members with HDL who have MetS and those who do not. According to these results, 48 patients showed an HDL of <40 mg/dl (mean ± SD = 39.35 ± 4.25). Out of 48 patients, 34 showed MetS with COPD with HDL (mean ± SD = 38.8 ± 3.8), and 14 patients showed COPD but no MetS with TG values (mean ± SD = 39.9 ± 4.7). When compared between these two, we did not observe a statistical difference (t = 0.8502; df = 46; p = 0.3996). Fifty-two patients in the present study showed HDL levels of >40 mg/dl (mean ± SD = 45.3 ± 11.5), of which 10 had MetS with COPD with a mean HDL value (mean ± SD = 44.7 ± 8.2) and 42 patients had COPD with no MetS with an HDL level (mean ± SD = 45.9 ± 6.7). When comparing patients with COPD who had an HDL of less than 40 mg/dl with those who did not.

**Table 6:** Low HDL and metabolic syndrome (MetS) patients in study population HDL: High-density lipoprotein Data presented in mean and standard deviations. p < 0.05 considered for statistical significance

Low HDL (<40 mg/dl)	MetS patients with COPD (mean ± SD)	Non-MetS patients with COPD (mean ± SD)	Total mean ± SD	p-value
Yes	38.8 ± 3.8	39.9 ± 4.7	39.35 ± 4.25	<0.0001
No	44.7 ± 8.2	45.9 ± 6.7	45.3 ± 11.5	

## Discussion

The present study aimed to determine the prevalence of MetS in cases of COPD and investigate its association with the severity of COPD. In a study by Mekov [12], the mean fasting blood glucose was higher than what we found in our study (102.4 ± 16.3 mg/dl). While the mean TG and HDL cholesterol levels in our sample were 164.2 ± 43.2 mg/dl and 43.1 ± 6.5 mg/dl, respectively, in research by Breyer [13], on MetS in COPD patients, the corresponding values were 136.04 mg/dl and 37. In comparison to Breyer [13], our study population had higher levels of HDL and TG. The mean Tc in our sample was 168.2 ± 44.3 mg/dl, while the mean LDL was 94.2 ± 46.4 mg/dl. We found that the mean LDL and Tc of the Sikter [14] study were lower than our investigation's results. In our sample cohort of COPD patients, 44% exhibited three or more components of MetS, indicating that MetS was prevalent in these individuals. Mekov's research [12] found the prevalence at 57%, a higher number than what we found in our study. However, the findings of a pooled review of the literature from PubMed and Embase, which included the original research, indicated that the prevalence was 34%, which was lower than the prevalence that we found in our analysis. Only two of the seven people who participated in our study were devoid of any MetS components, while the other seven individuals contained all five. Only one component of the MetS was present in 14 patients, whereas 30 people had two components. The 46 patients with one or both MetS components had a higher risk of cardiovascular disease and MetS in the future and required MetS screening. MetS incidence varied significantly among COPD stages (p < 0.05). Many global investigations have confirmed the conclusions above. We classified 64% of the participants in our study as belonging to the upper and lower socioeconomic classes as well as the lower socioeconomic classes (IV and V). In addition, 36% of the participants were from the lower middle class. One of the most significant risk factors for COPD is a low socioeconomic level, and the community that is the focus of this inquiry is a group that is a good match for this risk factor because of the prevalence of overcrowding and repeated infections of the respiratory tract. We found the prevalence of MetS to be 32%, 54%, and 14%, respectively, in classes III, IV, and V; however, none of these classes had a significant p-value greater than 0.05. The study's conclusions showed that whereas 59% of individuals with normal fasting glucose levels had MetS, 52% of COPD patients had abnormal fasting glucose levels. When a p-value is greater than 0.05, it means that the difference in the prevalence of MetS between COPD patients with impaired fasting glucose and those without it is not statistically significant. Conversely, there was no difference in the prevalence of MetS between COPD patients without impaired fasting glucose. Cebron [10] found a 66% prevalence of high fasting blood glucose in his research, but our evaluation found only a 50% prevalence, less than the previous study's findings. In contrast, Ghatas [15] determined the prevalence to be nearly identical to ours, at 42.60%. Meanwhile, Ali et al. [16] discovered that it was 57.1%. Breyer's [13] research revealed that hyperglycemia is the most common MetS component among COPD patients. Our results showed that in 31 patients (70%) with MetS and in 14 patients (30%) without MetS, there was a link between abdominal obesity and the existence of MetS. In addition, 13 people with MetS and COPD diagnoses did not have abdominal obesity. Patients with COPD who are abdominally obese have a significantly higher prevalence of MetS than those who are not (p = 0.05). In this regard, patients who are abdominally obese and those who are not differ. While the frequency was 51.10% in the research of Ghatas [15], it was only 49% in the study of Mekov [12]. Abdominal obesity is the most frequent component of MetS, which is rather common in COPD patients, according to research by Breyer [13], because individuals with COPD often have a very high frequency of illness. Our findings revealed that 28 of the 45% of COPD patients had MetS, and their BMI was above the recommended threshold. We identified only 16 individuals with BMIs greater than 25 as having MetS. There was a statistically significant difference (p < 0.05) in the prevalence of MetS between overweight COPD patients and those who were not. Ghatas [15] found that 29% of COPD patients with a BMI of less than 25 kg/m2 had MetS. In contrast, our study indicated that the prevalence was 61.7%, which is almost identical to the previously reported data, with a substantial difference between the two groups. Ten individuals did not have MetS; in contrast, 48% of the study group had low HDL levels, and 34 of those individuals had MetS. Patients with COPD who had higher HDL compared to those who did not show a statistically significant difference in the prevalence of MetS (p < 0.05). A study by Liu [17] found the prevalence of low HDL to be 70%, larger than our study group. In contrast, another study by Breyer [13] found it to be 35.7%, significantly lower than our study population. Compared to 30 patients who had increased TG levels and made up 42% of our research group, only 14 study participants who did not have elevated TG levels had MetS. Patients with COPD who have increased TG compared to those who do not have MetS are statistically significant (p < 0.05). While Mekov reported 46%, our study sample exhibited a 54% prevalence of elevated TG. Sonaullah [18] discovered 51.4%, which was comparable to our findings. According to Breyer [13], COPD patients with MetS had 226.7 mg/dl TG levels, whereas those without MetS had 158 mg/dl. There was a significant difference between the two groups. The 178.2 and 152.9 mg/dl results between the two groups showed a statistically significant difference, according to our analysis. Our analysis, which was quite comparable to the previously described research, revealed that the group of people with MetS had somewhat higher TG levels. The results of the research by Patel [19] show that the BMIs of COPD patients with MetS and those without it differ statistically significantly. People with MetS had BMIs of 29.2 kg/m2, whereas those without it had BMIs of 22.5 kg/m2. The results of our study showed a substantial difference between the two groups, with 27.4 and 24.2 kg/m2, respectively. Compared to the individuals in the previously referenced research, the group of MetS patients in our study had a lower BMI. In another study, Vujic [20] discovered that it was 30.0 and 24.88 kg/m2, respectively. This was larger than the population we used in our analysis and comparable to Breyer's research [13].

The results of Vujic [20] showed that the waist circumferences of COPD patients with MetS and those without the condition differed significantly, with the former group measuring 106.78 cm and the latter measuring 93.90 cm. The waist circumferences of the individuals in our sample ranged from 96.2 cm to 88.2 cm, demonstrating a significant difference between the two groups. This difference contradicts the findings of the previous research, as the lower socioeconomic class is associated with a higher percentage of underweight individuals. Research by Vujic [20] revealed a significant difference in the mean FBS of COPD patients with MetS compared to those without the condition. The mean results for the two groups were 94.59 mg/dl and 116.75 mg/dl, respectively. There was a significant difference between the two groups, as shown by Breyer's research [13], which indicated that the FBS of COPD patients with MetS was 115.3 mg/dl and the FBS of COPD patients without MetS was 92.9 mg/dl. Compared to other studies, we discovered that the FBS levels in our study population of people with MetS and those who did not have MetS were very different. There was a significant difference in FBS levels between the two groups (110.8 mg/dl and 98.2 mg/dl).

## Conclusions

Individuals with MetS were more likely to have high BP, raised TG, low HDL cholesterol, abdominal obesity, and other risk factors. A great deal of difference existed between the two groups. Therefore, we recommend that patients with COPD who also have diabetes, high BP, dyslipidemia, or reduced fasting glucose levels begin using insulin, lipid-lowering medications, and oral hypoglycemic medications. Our research demonstrates that patients with MetS exhibit elevated levels of VLDL, waist-to-hip ratio, total cholesterol, and waist circumference.
